# Influence of Drying Temperature on the Different Thermodynamic Parameters during the Indirect Convective Solar Drying of *Crocus sativus L.* Of Morocco Thin-Layer Solar Drying of Moroccan Saffron

**DOI:** 10.1155/2022/1656862

**Published:** 2022-05-19

**Authors:** Khadija Oubella, Hind Mouhanni, Younes Bahammou, Ali Idlimam, Abdelkader Lamharrar, Abdelaziz Bendou

**Affiliations:** ^1^Research Team Materials, Mechanical and Civil Engineering, ENSA, Ibn Zohr University, BP 1136, Agadir, Morocco; ^2^Laboratory of Solar Energy and Medicinal Plants, Cadi Ayyad University, BP 2400, Marrakesh, Morocco

## Abstract

This work deals with the study of the drying kinetics of *Crocus sativus* L. using convective solar drying. The main objective was to identify the influence of airflow drying temperatures for ambient air temperature ranged between 15.6 and 18.9°C, and a relative humidity between 24.4 and 46.5%. The equilibrium moisture content varies from 0.09 to 0.06 (% d.b), respectively, for drying air temperatures 35–50°C. The airflow velocity was about 0.2 m s^−1^, which implied establishing a phenomenological diffusion model of the water within the matrix. Empirical models were also determined as well as a polynomial equation (order 3) of the characteristic drying curve. The Midilli–Kucuk model was found to be the best to describe the experimental drying curves of *Crocus sativus* L. The effective moisture diffusivity ranged between 0.87 and 1.46 10^−11^ m^2^ s^−1^ for airflow temperature 35 and 50°C, while the average activation energy was calculated as 28.76 kJ mol^−1^. The increase in temperature decreases the total energy consumption which varies, respectively, from 3.211 to 2.681 kWh.

## 1. Introduction

*Crocus sativus* L. is the most expensive spice in the world, largely used for its biological virtues [1]. It is a plant from the Iridaceae family; its dried stigmas give the saffron used as herbal medicine, culinary spice, and for its coloring and flavoring characteristics [2]. The Mediterranean environment is considered the most adequate to produce saffron. Iran, Greece, Morocco, India, Spain, Lebanon, and Italy are the main producers of saffron in the world [3]. In Morocco, saffron is cultivated in a small region in the south named Taliouine, particularly in the Anti-Atlas Mountains [4].

Saffron quality is determined by three main metabolites: Crocine (C_44_H_64_O_24_) is responsible for saffron's color, picrocrocine (C_16_H_26_O_7_) is the principal cause for saffron's bitter taste, and safranal (C10H14O) is the most abundant volatile oil responsible for the aroma of saffron. The aglycone 4-hydroxy-2,6,6-trimethyl-1-cyclohexene-1-carboxaldehyde (HTCC) is liberated enzymatically by *β*-glucosidase action from the monoterpene glycoside picrocrocin and transformed to safranal by dehydration of the product during the drying process [4, 5]. The concentration of these components is affected by several factors such as harvesting conditions, drying temperature, and storage conditions [6].

In the agri-food sector, drying is an important tool to preserve perishable food, and medicinal and aromatic plants after harvesting. Drying is an operation involving phases of evaporation as the main way to remove water out from the pores. It allows reducing the free water contained in plants to prevent the development of microorganisms. Indeed, dry biomaterials are less prone to microbiological degradation in storage [7–9]. Drying can minimize the chances of chemical and biochemical degradation in the porous structure of the products, thus extending their shelf life [10]. Thus, it keeps seasonal plants available all year round to generally be used for culinary applications and treat many diseases [11].

To ameliorate the quality of dried products, researchers have developed many advanced drying techniques that can be used as stand-alone methods or combined with other drying techniques such as microwave-drying, vacuum-drying, freeze-drying, infrared-drying, conventional sun-drying, and convective solar-drying [12, 13]. Drying methods can be associated with some modern pre-treatment techniques: texturing by Instant Controlled Pressure-Drop DIC, plasma-assisted vacuum drying, etc. Freeze-drying has shown that low processing temperatures improved the sensorial and nutritional quality of dried products. Combined drying is considered the best way to reduce energy consumption and improve end product quality [7, 14].

Numerous studies of the techno-economic process have been carried out to optimize the drying operation of food products [15–17]. Solar energy is one of the best solutions proposed to overcome this challenge. It is one of the oldest routes to preserve food. It has also been recognized as an effective way to reduce the energy consumption as it uses a renewable energy source. However, direct solar drying with aerothermal parameters such as temperature and climatic conditions, cannot be controlled and generates many disadvantages as degradation and contamination of food by insects and microorganisms [18, 19]. In this context, indirect solar drying is recommended to improve the quality of dry products as reported by many authors in the literature [20–22]. The energy performance of the solar dryer can be evaluated in order to optimize and improve its operating system by detecting energy losses in the dryer chamber [23].

*Crocus sativus* L. is characterized by an inner structure depending on its chemical composition and a unique response toward the aerothermal drying parameters. Thus, it is necessary to determine the characteristic drying curve (CDC) and describe the drying kinetics. Solar drying is considered an adequate alternative technique to conserve *Crocus sativus* L. from spoilage during the storage process. The laboratory-scale results presented in this paper should be extrapolated to the industrial scale to design an industrial and a specific solar dryer for this biological plant while preserving or even increasing its commercial economic value. In a country like Morocco, where solar irradiation is great, using solar energy is quite beneficial [24, 25].

The purpose of the present study was to study the drying kinetics of *Crocus sativus* L. in a convective solar dryer, to transform all experimental data of drying to a global curve named the characteristic drying curve (CDC), and to determine the different thermodynamic parameters describing the mechanisms that occurred during the drying process of saffron.

## 2. Materials and Methods

### 2.1. Material

*Crocus sativus* L. used in the drying experiments was grown in the Taliouine region of Morocco (altitude 1734 m, latitude 30°33′56^″^ N, and a longitude of 7°36′06^″^ W), in November 2019. The stigmas of *Crocus sativus* L. have an average length of 2–3 cm. The initial water content was 0.14 (g of water/g d.b).

No pre-treatment has been prepared to the stigmas of *Crocus sativus* L.

### 2.2. Drying Procedure

**Figure 1:**
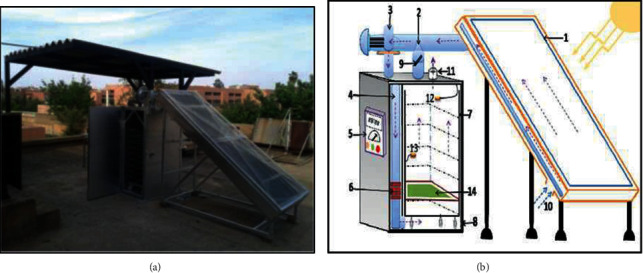
Schematic of the solar dryer used in the experiment: (1) solar collector; (2) circulation fan; (3) fan; (4) airflow direction; (5) control box; (6) auxiliary heating system; (7) shelves; (8) drying cabinet; (9) air recycling; (10) control foot; (11) exit of air; (12) humidity probes; (13) thermocouples; (14) sample holder.

The experimental study was carried out in Marrakech, Morocco using a forced convection solar dryer (Figure 1(a)), whose constituent elements are:

- A solar air collector of 2.5 m^2^ (2.5 m length and 1 m width) inclined with an angle of 30° with a cover of ordinary glass. The absorber of the solar air collector is fabricated from a black galvanized iron sheet, a thickness of 0.5 mm, with a nonselective surface.
- A circulation fan, an aeraulic suction pipe made up of a parallelepiped section tunnel, and a double *T* (consists of two interlocking *Ts*) that allows the total or partial recirculation of the air leaving the drying chamber. The double *T* has a butterfly valve to adjust the air flow.
- A drying cabinet has the following dimensions: 1.40 m height, 0.90 m depth, and 0.50 m width, and consists of 10 racks.
- A centrifugal fan, with an upstream throttling which allows to fix the air flow rate (varies from 0.028 to 0.0889 m^3^ s^−1^).
- A thermoregulator with a range of 0–99 ± 0.1°C, connected to a platinum probe (PT100) acting on the electric auxiliary heating (4 kW resistors), which allows to fix the temperature at the entrance of the drying chamber.
- Electrical resistors 4 kW acting as an auxiliary source of energy.

To control the drying parameters and conditions as relative humidity and temperature of the ambient air (Table 1) used during the drying experiment of *Crocus sativus* L. different apparatus were operated:

- Thermo-hygrometer, HI 9564 characterized by ABS body RH probe with a built-in microchip, temperature readout°C/F, and 250-hour battery life with a battery level indicator for determining the air flow relative humidity and ambient air temperature.
- Solar power meter, solar radiation measurement up to 1999 W m^−2^ or 634 BTU/(ft2 ∗ h). With the following characteristics: High accuracy and rapid response, data hold function to hold measurement values, unit and sign display for easy reading, measuring unit selection among W m^−2^ and BTU/(ft2 ∗ h). Manual scale selection, direct reading with no adjustments needed, maximum and minimum values, and low battery indication.
- Analytical balance, ANBA-4001 high precision with capacity 30 g/180 g ± 0.0001 g (0.1 mg).
- Thermal imaging camera, FLIR i5 Infrared Camera Thermal.

This dryer was developed and described by many researchers in several studies [26–28]. The drying process of *Crocus sativus* L. started by pruning the stigmas; the drying was performed with an airflow temperature of 35, 40, and 50°C using the solar collector assisted by the auxiliary heater for controlling the drying air temperature [29] using a constant rate of 300 m^3^ h^−1^ or about 0.2 m s^−1^ airflow speed.

The air is first heated in the solar collector and then led into the drying chamber (Figure 1(b) shows the direction of the air) where a heat transfer occurs from the air to the product, and a mass transfer of the product to air during drying.

The samples were weighed and placed on the first shelf of the drying cabinet. The mass of the stigmas used in the drying experiments was (0.99–1.00 g) ± 0.0001 g.

During each drying experiment, the weight of the product was measured by removing it from the drying cabinet for approximately 30 s. These measures M_h_(t) were undertaken each 5 min. As the mass decreases, this interval can expand to 10 or 15 min as soon as the mass became stable.

The dry mass (M_d_) of *Crocus sativus* L. is determined by total dehydration in the oven at 103°C for 16 h. The dry base moisture content at time *t* was defined using the following equation:

(1) $$\begin{array}{c} \text{X}\left(t\right)=\frac{{\text{M}}_{\text{h}}\left(t\right)-{\text{M}}_{\text{d}}}{{\text{M}}_{\text{d}}}. \end{array}$$

### 2.3. Determination of the Characteristic Drying Curve (CDC)

The final moisture content or the equilibrium moisture content is considered as the optimal water content for which the product does not deteriorate [30]. Therefore, its determination at the end of the drying experiment is important to describe the kinetics of drying.

Studying the kinetics of drying was carried out by determining the characteristic drying curve (CDC). D.A. Van Meel [31] developed a method that consists of using the initial moisture content X_0_ with the equilibrium moisture content Xe (obtained from the isotherms of sorption) to normalize the moisture ratio MR (t) and the dimensionless drying rate (f), as calculated in the following equations:

(2) $$\begin{array}{c} \text{MR}\left(\text{t}\right)=\frac{\text{X}\left(\text{t}\right)-{\text{X}}_{\text{e}}}{{\text{X}}_{\text{0}}-{\text{X}}_{\text{e}}},\\ \text{f}=\frac{{\left(-\text{dX}/\text{dt}\right)}_{\text{t}}}{{\left(-\text{dX}/\text{dt}\right)}_{\text{0}}}. \end{array}$$

The characteristic drying curve (CDC) is given by *f* = *f* (MR). “OriginPro 9.0” software was used to estimate it and find the best polynomial equation for *Crocus sativus* L.

### 2.4. Modelling of Solar Drying Curves

#### 2.4.1. Diffusion Controlled Process

A phenomenological analysis of the operation was achieved by [32]. A negligible external resistance (NER = 0) allows the drying to become completely controlled by the internal water diffusion. This hypothesis can be respected when the airflow velocity is higher than the critical airflow velocity, CAV, as defined by Nguyen et al. [33].

(3) $$\begin{array}{c} \text{CAV}=2.33{\left(\left(\frac{\mu^{\left(0.56-\left(1/3\right)\right)}}{\rho_{v}{}^{\left(0.56+\left(2/3\right)\right)}}\right)\frac{L^{0.44}}{D_{\text{vapor}-\text{air}}^{2/3}}\frac{P_{\text{total}}D_{\text{eff}}{\left(-d\rho_{w}/dz\right)}_{z=z_{\text{surface}}}}{P_{{\text{w,T}}_{\text{s}}}a_{\text{w,s}}-P_{\text{w,air}}}\right)}^{1.78}. \end{array}$$

In our case of stigmas of *Crocus sativus* L., CAV was estimated from the following data: 0.87 10^−11^ to 1.46 10^−11^ (see Table 2)

#### 2.4.2. Fitting Models

A curve-fitting method was used to solve nonlinear least squares problems based on modeling of drying curves. It allows us to find a most adequate equation that can express the experimentally reduced moisture content as a function of time MR = *f*(*t*).

In the literature, many empirical and semi-empirical models were applied to describe the thin layer solar drying curve of aromatic and medicinal plants [15, 21]. Each model is characterized by a unique formula including specific coefficients.

In this work, eight thin-layer drying models were used to describe the experimental data. The appropriate model is chosen according to the following criteria: the highest correlation coefficient (*r*) and the lowest SSE [34]:

(4) $$\begin{array}{c} r=\sqrt{\frac{\sum_{i=1}^{N}{\left(MR_{eq_{\text{i,pred}}}-{\bar{MR}}_{eq_{\text{i,exp}}}\right)}^{2}}{\sum_{i=1}^{N}{\left(MR_{eq_{\text{i,exp}}}-{\bar{MR}}_{eq_{\text{i,exp}}}\right)}^{2}}},\\ SSE=\sqrt{\sum_{i=1}^{N}\frac{{\left(MR_{{\text{eq}}_{\text{i,exp}}}-MR_{{\text{eq}}_{\text{i,pred}}}\right)}^{2}}{f}}. \end{array}$$

MR_i,pred_: i^ème^ moisture ratio predicted by model.

MR_i,exp_: i^ème^ experimental moisture ratio.

N : Number of experimental data.

The CurveExpert Professional software was used to determine the characteristic coefficients of each model and its statistical parameters for the three temperatures. This program is based on the nonlinear optimization method Marquard–Levenberg [35]. It also allowed extracting the best model explaining the kinetics of drying of *Crocus sativus* L.

### 2.5. Effective Diffusivity and Activation Energy

By assuming the external resistance (Negligible External Resistance NER = 0) is negligible, based on the airflow speed higher than the CAV value, drying is controlled by the diffusion of water within the matrix. This transport of moisture from the inside to the product surface is accorded to Fick's second law [24, 36]:

(5) $$\begin{array}{c} \frac{\partial MR}{\partial t}=D_{\text{eff}}\nabla^{2}MR, \end{array}$$

where *D*_*eff*_ is the effective moisture diffusivity (m^2^·s^−1^).

The analytical solution of this equation is given by the equation developed by Crank in [37] for slab geometry. In the case of an infinite plate:

(6) $$\begin{array}{c} \text{MR}\left(t\right)=\frac{8}{\pi^{2}}\sum_{n=0}^{\infty }\frac{1}{{\left(2n+1\right)}^{2}}\text{exp}\left[-{\left(2n+1\right)}^{2}\frac{\pi^{2}D_{eff}\cdot t}{4L^{2}}\right]. \end{array}$$

When the drying time is long, this solution can be expressed as follows [38]:

(7) $$\begin{array}{c} MR\left(t\right)≅\frac{8}{\pi^{2}}\exp\left[-\frac{\pi^{2}D_{\text{eff}}.t}{4L^{2}}\right]. \end{array}$$

L (*m*) is the half-thickness of the used samples.

Equaton (7) can be written in a logarithmic form:

(8) $$\begin{array}{c} Ln\left(MR\right)=Ln\left(\frac{8}{\pi^{2}}\right)-\left(\frac{\pi^{2}D_{\text{eff}}.t}{4L^{2}}\right). \end{array}$$

The effective diffusivity (D_eff_) was calculated using the plot slope B of the straight line corresponding to ln (MR) = *f*(*t*).

(9) $$\begin{array}{c} \text{B}=\left(-\frac{\pi^{2}{\text{D}}_{\text{eff}}}{{4\text{L}}^{2}}\right). \end{array}$$

Activation energy E_a_ (kJ·kg^−1^) can be defined as the energy necessary to initiate the drying process and other physical phenomena of a system [38, 39].

It was deduced from the law of Arrhenius, using the same procedure as the diffusion coefficient [36, 40].

(10) $$\begin{array}{c} {\text{D}}_{\text{eff}}{=\text{D}}_{\text{0}}\text{exp}\left(-\frac{{\text{E}}_{\text{a}}}{\text{RT}}\right), \end{array}$$

(11) $$\begin{array}{c} \text{B}=\left(-\frac{{\text{E}}_{\text{a}}}{\text{R}}\right), \end{array}$$

where B is the slope of the straight line corresponding to Ln (D_eff_) versus 1/T (the inverse of temperature in K) and D_0_ is the Arrhenius factor in m^2^ s^−1^, R is the universal gas constant in *J* mol^−1^ K^−1^, and T is the temperature.

### 2.6. Energy Consumption in Solar Drying

#### 2.6.1. Total Energy Consumption

Total energy consumption at different air temperatures in a convective solar dryer is considered as the sum of mechanical and thermal energies (kWh), which is estimated using equation (12) [41]:

(12) $$\begin{array}{c} {\text{E}}_{\text{t}}{=\text{E}}_{\text{ther}}{+\text{E}}_{\text{mec}}. \end{array}$$

E_ther_ represents the thermal energy consumed (kWh) at different air temperatures; it was calculated from the following equation [42]:

(13) $$\begin{array}{c} E_{\text{ther}}=A\cdot \nu \cdot \rho_{a}\cdot C_{a}\cdot \Delta T\cdot D_{t}, \end{array}$$

where

*A* is the surface area (m^2^) of the tray in which the sample is placed, *ν* is the air flow rate (m s^−1^), *ρ*_*a*_ is the air density (kg·m^−3^), C_a_ is the specific heat of dry air at known temperature (kJ kg^−1^ C^−1^), Δ*T* represents the temperature difference between air drying and ambient temperature (°C), and D_*t*_ is the total drying time (*h*) for each air temperature.

C_a_ and *ρ*_a_ were calculated as a function of the drying air temperature (*K*) using equations (14) and (15), [42–44]:

(14) $$\begin{array}{c} \rho_{a}=\frac{101\cdot 325}{0\cdot 287\times T}, \end{array}$$

(15) $$\begin{array}{c} C_{a}=1\cdot 04841-\frac{3\cdot 83719\times T}{10^{4}}+\frac{9\cdot 45378\times T^{2}}{10^{7}}-\frac{5\cdot 49031\times T^{3}}{10^{10}}+\frac{7\cdot 92981\times T^{4}}{10^{14}}. \end{array}$$

The mechanical energy consumed is defined as the electrical energy consumed during each drying experiment by the fan and the auxiliary heater; it is determined using equation (16) [41, 45]:

(16) $$\begin{array}{c} {\text{E}}_{\text{mec}}=\Delta \text{P}\cdot {\text{m}}_{\text{air}}\cdot {\text{D}}_{\text{t}}. \end{array}$$

ΔP is the pressure difference (mbar); mair is the inlet air mass (kg); Emec was measured in this study by an electric energy meter with an accuracy of 0.01 kWh.

#### 2.6.2. Specific Energy Consumption (SEC)

Specific energy consumption (kWh·kg^−1^) is defined as the amount of energy required to evaporate a unit mass of water from the product as shown in equation (17) reported by [41, 46].

(17) $$\begin{array}{c} \text{SEC}=\frac{{\text{E}}_{\text{t}}}{{\text{m}}_{\text{w}}}, \end{array}$$

m_w_ is the mass of the removed moisture (kg); it was determined by equation (18) [45].

(18) $$\begin{array}{c} {\text{m}}_{\text{w}}=\frac{{\text{w}}_{\text{0}}\left({\text{M}}_{\text{0}}-{\text{M}}_{\text{f}}\right)}{100-{\text{M}}_{\text{f}}}, \end{array}$$

where

ⱳ_0_ is the initial weight of the sample (kg), M_0_ is the initial moisture content (% d.b) at time *t* = 0, and M_f_ is the final moisture content (% d.b).

#### 2.6.3. Energy Efficiency

Energy efficiency *η*_*e*_ is considered as one of the parameters used to evaluate the dryer efficiency. In the literature, it can be derived using the balance equations for thermodynamic analysis [47]. Equation (19) was used to determine it [48]:

(19) $$\begin{array}{c} \eta_{\text{e}}=\frac{{\text{Q}}_{\text{w}}}{{\text{E}}_{\text{T}}}, \end{array}$$

where Q_w_ (kJ) represents the necessary energy for the evaporation of the moisture contained in the product. It was calculated using the following equation [45][48]:

(20) $$\begin{array}{c} {\text{Q}}_{\text{w}}{=\text{h}}_{\text{fg}}\cdot {\text{m}}_{\text{w}}. \end{array}$$

This relation is valid for materials with high moisture contents as declared by Muthu and Chattopadhyay in [49].

There are two expressions for the heat of vaporization (kJ kg^−1^) as a function of the drying air temperature [45]:

(21) $$\begin{array}{c} h_{\text{fg}}=2\cdot 503\times 10^{6}-2\cdot 386\times 10^{3}\times \left(T-273.16\right)273\cdot 16\leq T\left(K\right)\leq 338\cdot 72,\\ h_{\text{fg}}={\left(7\cdot 33\times 10^{12}-1\cdot 60\times 10^{7}\times T^{2}\right)}^{0.5}338\cdot 72\leq T\left(K\right)\leq 533\cdot 16. \end{array}$$

## 3. Results and Discussion

### 3.1. Drying Curves of *Crocus sativus* L

**Figure 2:**
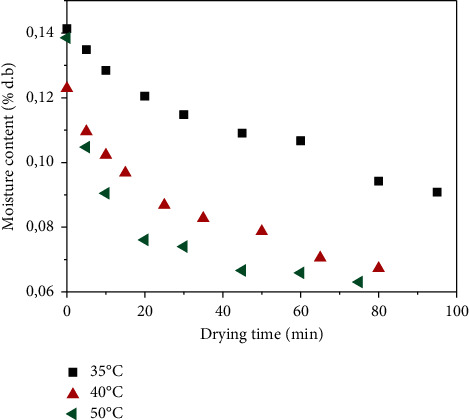
Variation of moisture content as a function of drying time for three temperatures of *Crocus sativus L.*

The operating parameters of airflow temperature and speed have a direct influence on the drying kinetics of *Crocus sativus* L. The drying time can be defined as the time required for a product to reach the final equilibrium moisture content called optimum moisture content from which a product does not deteriorate. Many studies in the literature have evaluated the effect of temperature on the drying time [30, 39]; it has been confirmed that the drying time decreases when the temperature increases.  Figure 2 represents the evolution of the moisture content depending on the drying time at the three temperatures (35, 40, and 50°C). The higher the airflow temperature, the quicker the decrease in the moisture content.

**Figure 3:**
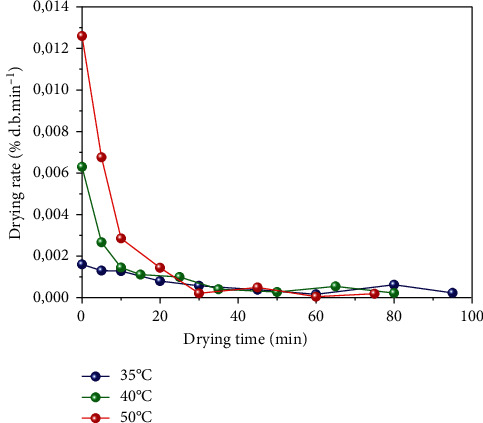
Evolution of drying rate versus time for different drying air temperatures of *Crocus sativus L.*

The variation of the drying rate versus the drying time is shown graphically in Figure 3. It is observed that the drying rate of *Crocus sativus* L. decreases when the drying temperature decreases.

In the literature, the drying curves are divided into three periods during the drying process. In period 0 (transitory phase), an increase in the temperature of the product apparently by the heat transfer process is observed; thus, the product has the drying air temperature. Also, the mass transfer process is established upon the surface of the product that marks the beginning of period 1. Period 1 (drying rate constant) is characterized by the presence of free moisture on the product's surface where the vapor pressure on the surface is equal to the saturation vapor pressure. It lasts as long as the surface is supplied with free water. During period 2 (decreasing drying rate), the vapor pressure becomes lower than the saturated vapor pressure, and the moisture content decreases.

In our experiment conditions, there was an absence of phases 0 and 1. Only phase 2 existed in the drying curves of *Crocus sativus* L., and similar results were observed in other works [39, 50].

**Figure 4:**
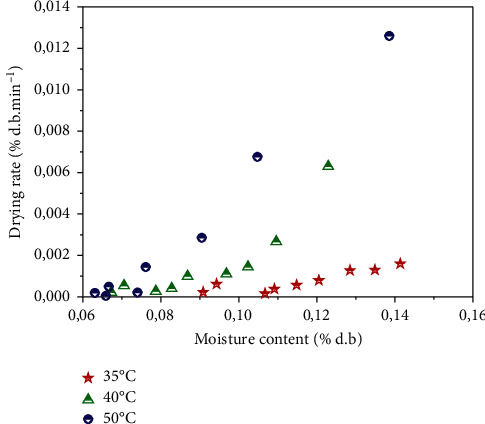
Evolution of drying rate versus moisture content for three temperatures of *Crocus sativus* L.

In addition to these drying curves, the evolution of the drying rate as a function of the moisture content is given in Figure 4. The drying rate varies proportionally with the moisture content, which means that the drying rate increases with the moisture content. The highest values of the drying rate were observed at 50°C. These results are confirmed by other scientific works [50].

### 3.2. Characteristic Drying Curve (CDC) of *Crocus sativus* L

The aim of this section is focused to transform all experimental data into a single saffron drying curve called the characteristic drying curve (CDC) of *Crocus sativus* L.

**Figure 5:**
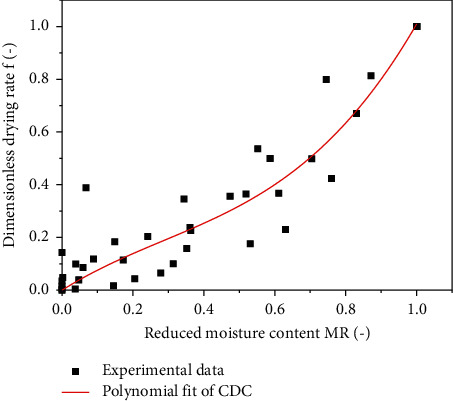
Characteristic drying curve of *Crocus sativus L.*

The determination of this curve is important. Indeed, it can be possible to describe the drying kinetics of *Crocus sativus* L. at any condition of the drying air by knowing the values of the initial water content and that of equilibrium (which are deduced from the sorption isotherms). From a point of view of the solar dryer sizing, it is interesting to elaborate the characteristic drying curve for the present product. Thus, the CDC model can valorize all experimental data and can be exploited not only by the experimenter but also by the engineering community [51, 52].  Figure 5 shows the characteristic drying curve of *Crocus sativus* L. where all experimental data are grouped. Different mathematical correlations based on the nonlinear optimization method Marquard–Levenberg were used to determine the equation adequate for describing the drying kinetics of *Crocus sativus* L. (*f* = *f*(MR)). A polynomial fit (order 3) was found as the best equation to describe the characteristic drying curve of *Crocus sativus* L.

The equation *f = f*(*MR*) is in the form of a polynomial with degree 3:

(22) $$\begin{array}{c} f=0\cdot 8441MR-0\cdot 9840{\text{MR}}^{2}+1\cdot 1495{\text{MR}}^{3},\\ r=0\cdot 9414,\\ \text{SSE}=0\cdot 4193. \end{array}$$

### 3.3. Fitting of the Drying Curves of *Crocus sativus* L

The moisture ratio values of *Crocus sativus* L. obtained from measuring the moisture content at three drying air temperatures were represented as a function of the drying time. The drying curves were fitted by eight statistical models found in the literature.  Table 3 shows the coefficients of each model at different temperatures with the statistical analysis. The best smoothing is chosen based on two important statistical parameters which have the highest correlation coefficient (*r*) and the lowest sum squared estimate of errors (SSE) [53].

**Figure 6:**
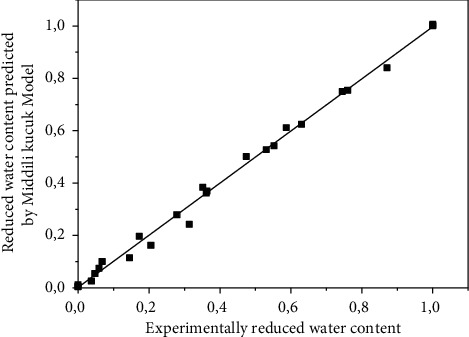
The values of MR predicted by the Middili–Kucuk model as function of its experimental data.

Table 3 allowed finding the Midilli–Kucuk model as the most suitable model for describing the thin drying curves of *Crocus sativus L.* with a correlation coefficient greater than 0.99 and an SSE lower than 0.02. A good agreement was obtained between the experimental data and the values predicted by the Midilli–Kucuk model for moisture ratio (Figure 6).

To well-define the evolution of the four coefficients a, *k*, n, and *b* of the Midilli–Kucuk model, equation versus airflow temperature can be modeled as a function of the drying air temperature as follows:

(23) $$\begin{array}{c} \text{MR}\left(t\right)=a\;\exp\left(-kt^{n}\right)+\text{bt,} \end{array}$$

where:

(24) $$\begin{array}{c} a=1\cdot 1209-0\cdot 0053T+6\times 10^{-5}T^{2}\;r=1, \end{array}$$

(25) $$\begin{array}{c} \text{b}=-\text{0.}04\;+\text{0.}0016\text{T}-2\;\times 10{\text{.}5}^{\mathrm{-5}}{\text{T}}^{2}\;\text{r }=\;1, \end{array}$$

(26) $$\begin{array}{c} k=0.3152-0.0191T+3\times 10^{-4}T^{2}\;r=1, \end{array}$$

(27) $$\begin{array}{c} n=-1.3166+0.0915T-0.001T^{2}\;r=1, \end{array}$$

(28) $$\begin{array}{c} r=0\cdot 9975, \end{array}$$

Using equations (24)– (27), it was easy to determine with significant accuracy the moisture ratio MR at airflow drying temperatures of 35, 40, and 50°C for *Crocus sativus* L.

### 3.4. Determination of Effective Moisture Diffusivity and Activation Energy

**Figure 7:**
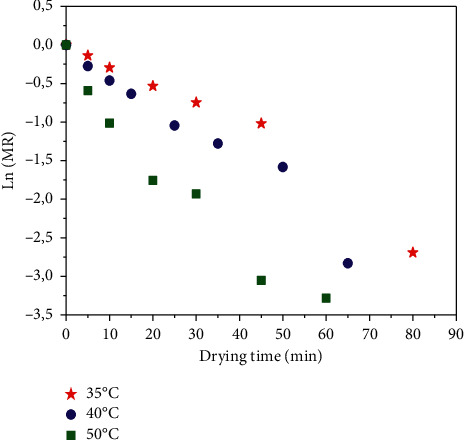
Plot of ln (MR) versus drying time for different drying air temperatures.

To describe the mass transfer process during the drying of *Crocus sativus* L., it is indispensable to determine the effective diffusivity at different drying airflow temperatures using Fick's second law as detailed in (5) and (9) Table 4 shows the effective diffusivity values at the three air temperatures, which were obtained from the graphs Ln (MR) = *f*(*t*) (Figure 7). It is observed that D_eff_ increases with an increase in drying air temperatures. These results correlated the previous studies that exist in the literature for other products [26, 54, 55].

The D_eff_ values found for *Crocus sativus* L. varied between 0.87 10^−11^ and 1.46 10^−11^ m^2^ s^−1^. These values respect the overall margin 10^−8^ to 10^−12^ m^2^ s^−1^ for D_eff_ of food products [39].

**Figure 8:**
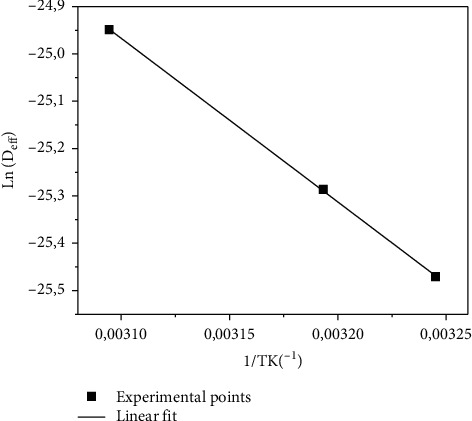
Influence of the drying air temperature on the effective moisture diffusivity of Crocus sativus L.

The activation energy was calculated from (10) and (11) based on Figure 8 which represents the natural logarithm of D_eff_ as a function of the inverse of the drying air temperature. The value of the activation energy was 28.76 kJ mol^−1^ for *Crocus sativus* L.

### 3.5. Energy Consumption in Solar Drying

#### 3.5.1. Total energy consumption / Energy efficiency

**Figure 9:**
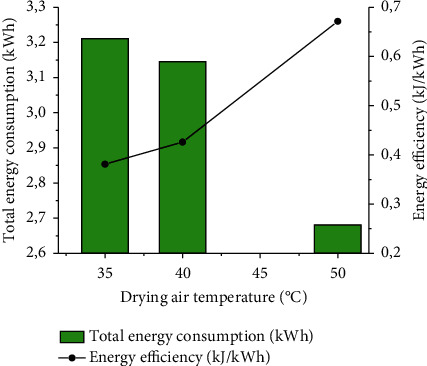
The variation of the total energy consumption and the energy efficiency as depending on the drying air temperature.

The figure below (Figure 9) displays on one hand the evolution of the total energy consumption and on the other the variation of the energy efficiency in a forced convection solar drying of *Crocus sativus* L. all as a function of the drying airflow temperature.

It is noted that the total energy consumption decreases with an increase in temperature, where the values were 3.211, 3.145, and 2.681 kWh at 35, 40, and 50°C, respectively. This observation can be explained by the fact that the total energy consumption depends on the drying time (equations (13) and (14)). In other words, when the temperature decreases, the total energy consumption increases with drying time.

Moreover, it was observed that the energy efficiency increases with an increase in the airflow temperature. Thus, the energy efficiency varies contrary to the total energy consumption, which verifies equation (20). Its values are represented with a minimum value of 0.01% found at 35°C and a maximum of 0.02% at 50°C.

To increase the efficiency of the solar dryer, it was necessary to minimize the total energy consumption by reducing the consumption of electrical energy. In this context, numerous researchers have developed methods that can increase the efficiency of solar dryers. For example, Yassen and Al-Kayiem encouraged the use of the recovery dryer [56]. Murugan et al. have used corrugated booster reflectors (CBR) [57]. Eltawil et al. proposed the combination of solar PV systems with a solar tunnel dryer (STD) using a thermal curtain [58].

These results are in line with other studies on solar drying of many agri-food products such as horehound leaves, potato chips, sweet cherry, and black ginger, as reported, respectively, by [50, 58–60].

#### 3.5.2. Specific Energy Consumption

**Figure 10:**
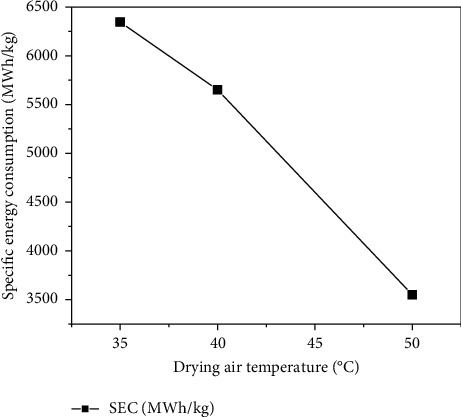
The evolution of the SEC versus drying air temperature.

The evolution of specific energy consumption as a function of temperature is shown in Figure 10. The values of SEC obtained were between 3550 and 6347 MWh/kg for drying air temperatures between 35 and 50°C. It is deduced from this graph that the SEC decreased as the drying air temperature increased. It was the same result as the total energy consumption. Therefore, the SEC varies proportionally with the total energy consumption and contrary with the energy efficiency, as illustrated in equation (18). This is due to the fact that increasing the drying temperature decreases the drying time.

These results are in agreement with other studies on solar drying of peppermint leaves and berberis fruit [42, 61].

## 4. Conclusion

The use of an indirect forced convection solar dryer for drying of the Moroccan saffron has proved that solar drying stays an efficient method for better conservation of aromatic and medicinal herbs.

The obtained results can be summarized as follows:

- At equilibrium, the increase in temperature (35–50°C) decreases the water content which varies, respectively, from 0.09 to 0.06 (% d.b).
- At *t* *=* *0*, the increase in temperature increases the drying rate which varies, respectively, from 0.0016 to 0.013 (% d.b.min^−1^).
- The characteristic drying curve (CDC) was found to be a polynomial equation (order 3) that allows describing the drying kinetics of *Crocus sativus* L. at other conditions of drying air.
- Midilli–Kucuk model was chosen as the most suitable equation for describing the drying behavior of *Crocus sativus* L.
- The maximum allowable drying temperature for *Crocus sativus* L. is 50°C.
- The effective moisture diffusivity varied between 0.87 and 1.46 10^−11^ m^2^ s^−1^ in the temperature range of 35–50°C.
- The activation energy was estimated to be 28.76 kJ mol^−1.^
- The energy efficiency and the specific energy consumption increase with an increase in the airflow temperature. Their values are represented, respectively, with a minimum value of 0.01% and 3550 MWh kg^−1^ found at 35°C, and a maximum of 0.02% and 6347 MWh·kg^−1^ at 50°C.

Finally, solar energy is considered as an effective renewable and alternative source to dry aromatic and medicinal plants, especially *Crocus sativus* L. This paper allows establishing the characteristic drying curve equation from the drying experimental data. This equation is necessary for simulating a solar drying and sizing and dimensioning it for the specific case of *Crocus sativus* L. to reach an adequate and professional solar dryer.

## Figures and Tables

**Table 1:** Drying parameters and conditions during experiments in the solar dryer.

Exp No	Airflow drying temperature (°C)	Airflow relative humidity (%)	Ambient temperature (°C)	t (min)
1	35	46.47	18.89	95
2	40	24.40	15.61	80
3	50	41.46	17.75	75

**Table 2:** Physical data for estimating the CAV value of *Crocus sativus* L. stigmas.

*a*_*w,s*_: Initial water activity at the surface = 1	1	—
*D*_eff_: Effective diffusivity (m^2^ s^−1^): Hypothesis of 1 ∗ 10^−11^ m^2^ s^−1^	1.00 *E*^−11^	m^2^ s^−1^
*L* : Length of stigmas, parallel to air flow (m): 0.3 mm	0.0003	m
*P*_total_: Ambient pressure (Pa): 1.00*E* + 5 Pa	1.00 *E*^+05^	Pa
*P*_w,air_: Vapor pressure of airflow (Pa) at about 40°C	7500	Pa
*z*: Position within the food (m)	1.00 *E*^−04^	m
*P*_*v*_: Density of vapor (kg·m^−3^)	0.69170059	kg·m^−3^
*P*_*w*_: Initial apparent density of water in the material (kg·m^−3^)	8.00 *E*^+02^	kg·m^−3^
*D*_vapor−air_: Diffusivity of water vapor (0.275 cm^2^ s^−1^)	2.75 *E*^−05^	m^2^ s^−1^
*μ*: Dynamic viscosity of the fluid (kg m^−1^ s^−1^): 1.3 E^−5^ Pa s	1.30 *E*^−05^	Pa·s
Thus, CAV (critical airflow velocity) became	2.25 *E* ^−03^	m·s^−1^

**Table 3:** Statistical parameters for each model according to the drying temperature.

Model name	T°(C)	Coefficients	*r*	SSE
Approximation of diffusion MR=aexp(−kt)+(1 − a)exp(−kbt)	35	*a* = 0.274919 *k* = 0.025321 *b* = 1.000016	0.987062	0.063674
	40	*a* = 1.0000 *k* = 0.040771 *b* = 1.0000	0.992494	0.046991
	50	*a* = 1.0000 *k* = 0.096969 *b* = 1.0000	0.990378	0.056035
Henderson and Pabis MR=*a* exp(−*kt*)	35	*a* = 0.989186 *k* = 0.024968	0.987190	0.058659
	40	*a* = 0.961574 *k* = 0.038664	0.993851	0.039391
	50	*a* = 0.966113 *k* = 0.092563	0.991124	0.049139
Logarithmic MR=aexp(−kt)+c	35	*a* = 1.182757 *k* = 0.016349 *c* = −0.223845	0.991433	0.051868
	40	*a* = 0.967432 *k* = 0.037812 *c* = −0.008219	0.993877	0.042458
	50	*a* = 0.936275 *k* = 0.108052 *c* = 0.045578	0.994877	0.040935
Midilli–Kucuk MR=aexp(−kt^*n*^)+bt	35	*a* = 1.007054 *k* = 0.057324 *n* = 0.633474 *b* = −0.003757	0.995431	0.041535
	40	*a* = 1.002236 *k* = 0.087317 *n* = 0.707525 *b* = −0.001741	0.998167	0.025477
	50	*a* = 1.001182 *k* = 0.197427 *n* = 0.702230 *b* = −0.000076	0.999027	0.019962
NewtonMR=exp(−kt)	35	*k* = 0.025322	0.987062	0.055143
	40	*k* = 0.040771	0.992494	0.040696
	50	*k* = 0.096969	0.990378	0.047358
Two-term MR=aexp(−k_0_t)+bexp(−k_1_t)	35	*a* = −17.719848 *k*_0_ = 0.034255 b = 18.696468 *k*_1_ = 0.033627	0.987778	0.067806
	40	*a* = 0.098095 k_0_ = 0.595591 b = 0.901910 *k*_1_ = 0.035569	0.995931	0.037933
	50	*a* = 0.544158 k_0_ = 0.209609 b = 0.455690 *k*_1_ = 0.045289	0.998798	0.022187
Wang and Singh MR=1+at+bt^2^	35	*a* = −0.018784 b = 0.000091	0.984337	0.064818
	40	*a* = −0.029208 b = 0.000219	0.971593	0.084190
	50	*a* = −0.043317 b = 0.000422	0.876853	0.177706
Page MR=exp(−kt^n^)	35	*k* = 0.025075 *n* = 1.002645	0.987063	0.058948
	40	*k* = 0.060268 *n* = 0.878508	0.995140	0.035032
	50	*k* = 0.193067 *n* = 0.712913	0.998780	0.018256

**Table 4:** Values of the effective diffusivity for three drying air temperatures.

	The slope	L (mm)	D_eff_ (m^2^ s^−1^)	*r*
35°C	−0.0321	0.2	8.67 10^−12^	0.9619
40°C	−0.0386	0.2	1.04 10^−11^	0.9570
50°C	−0.0541	0.2	1.46 10^−11^	0.9539

## Data Availability

The data used to support the findings of this study are available from the corresponding author upon request.
